# R2* for myocardial iron quantification at 3T CMR using an advanced shimming algorithm

**DOI:** 10.1186/1532-429X-15-S1-P38

**Published:** 2013-01-30

**Authors:** Gabriel C Camargo, Tamara Rothstein, Flavia P Jjunqueira, Daniel C Quintella, Ralph Strecker, Andreas Greiser, Joao A Lima, Patricia B Rizzi, Maria Eduarda Derenne, Ronaldo SL Lima, Ilan Gottlieb

**Affiliations:** 1 CDPI - Clínica de Diagnóstico por Imagem, Rio de Janeiro, Brazil; 2 Siemens LTDA, São Paulo, Brazil; 3 Siemens Healthcare, Erlangen, Germany; 4 Cardiology, Johns Hopkins University, Baltimore, MD, USA

## Background

Quantification of myocardial iron overload is critical for the management of patients with hemochromatosis. T2* relaxation time or its inverse R2*, are currently the non-invasive gold standard at 1.5T, as they strongly correlate with myocardial iron concentration, and are obtained using a fast single breath-hold ECG-gated multi-echo GRE sequence. However, due to high sensitivity to B0 inhomogeneities, R2* (1/T2*) quantification at 3T is still challenging. We aimed to evaluate if a recently developed advanced shimming protocol based on a GRE double-echo fieldmap acquisition, can improve the performance of R2* quantification at 3T.

## Methods

A total of 15 normal volunteers and 7 hemochromatosis patients (with a myocardial T2* measure <20 ms in the last 2 years, five of these on iron chelating therapy) were scanned at 1.5T and 3T (MAGNETOM Symphony and Verio respectively, Siemens, Erlangen, Germany) using breath-hold multi-echo GRE sequences, under standard entire FOV shimming (Protocol1). The same sequence was repeated at 3T after a double-echo GRE fieldmap guided a novel 3D shimming with the shim volume limited to the heart (Protocol2). All ROIs were placed at mid-interventricular septum, carefully avoiding the blood pool and all analyses were blinded.

## Results

**Figure 1:**
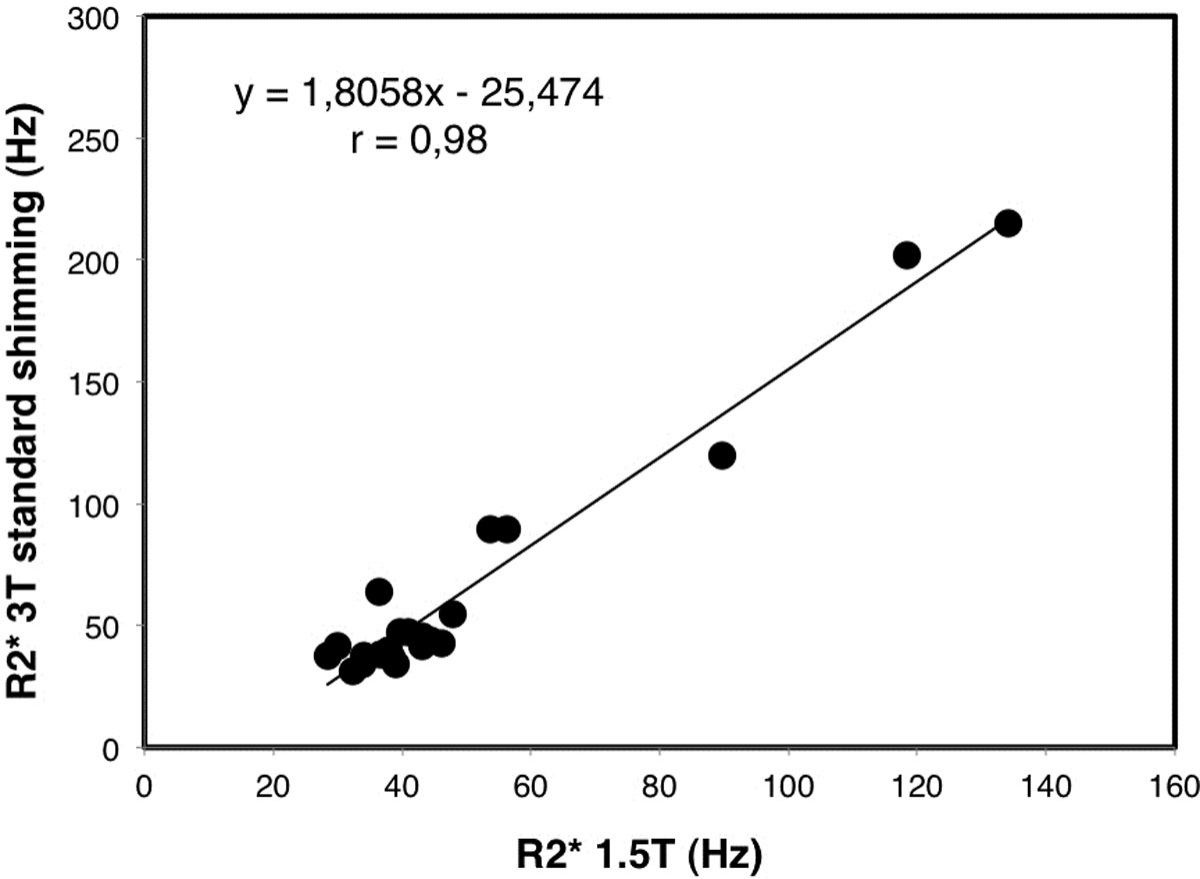
Linear regression between R2* measurements at 3T with standard shimming and R2* at 1.5T

**Figure 2:**
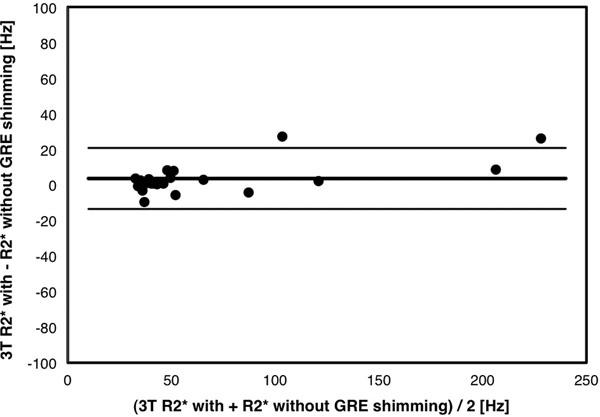
Bland-Altman plot of the R2* measurements at 3T performed with and without GRE shimming.

All patients were at sinus rhythm and their MRI scans had diagnostic image quality. Mean myocardial R2* values were not statistically different between protocols 1 and 2 (65.2±51.3 Hz and 68.6±56.8 Hz respectively, p=0.83). R2* in both 3T protocols showed strong linear correlation with R2* at 1.5T, with r=0.98 in both cases (Figure 1). R2* measurements with and without advanced shimming had excellent correlation with each other (r=0.99), with small bias (mean difference of 3.5±8.6 ms) as shown in Figure 2.

## Conclusions

In sustained shimmed 3T systems, R2* quantification can be reliably performed using standard shimming protocol, in strong correlation with 1.5T reference values. Our results do not show improvement in performance of R2* measurement using a new volume targeted shimming algorithm developed for 3T.

## Funding

Internal

